# Supplementary material for: Misfolded polypeptides are selectively recognized and transported toward aggresomes by a CED complex
Source: Nat Commun. 2017 Jun 7;8:15730. doi: 10.1038/ncomms15730 (PMC5467238; doi:10.1038/ncomms15730)
Supplement: Supplementary Information — Supplementary figures. [file ncomms15730-s1.pdf]

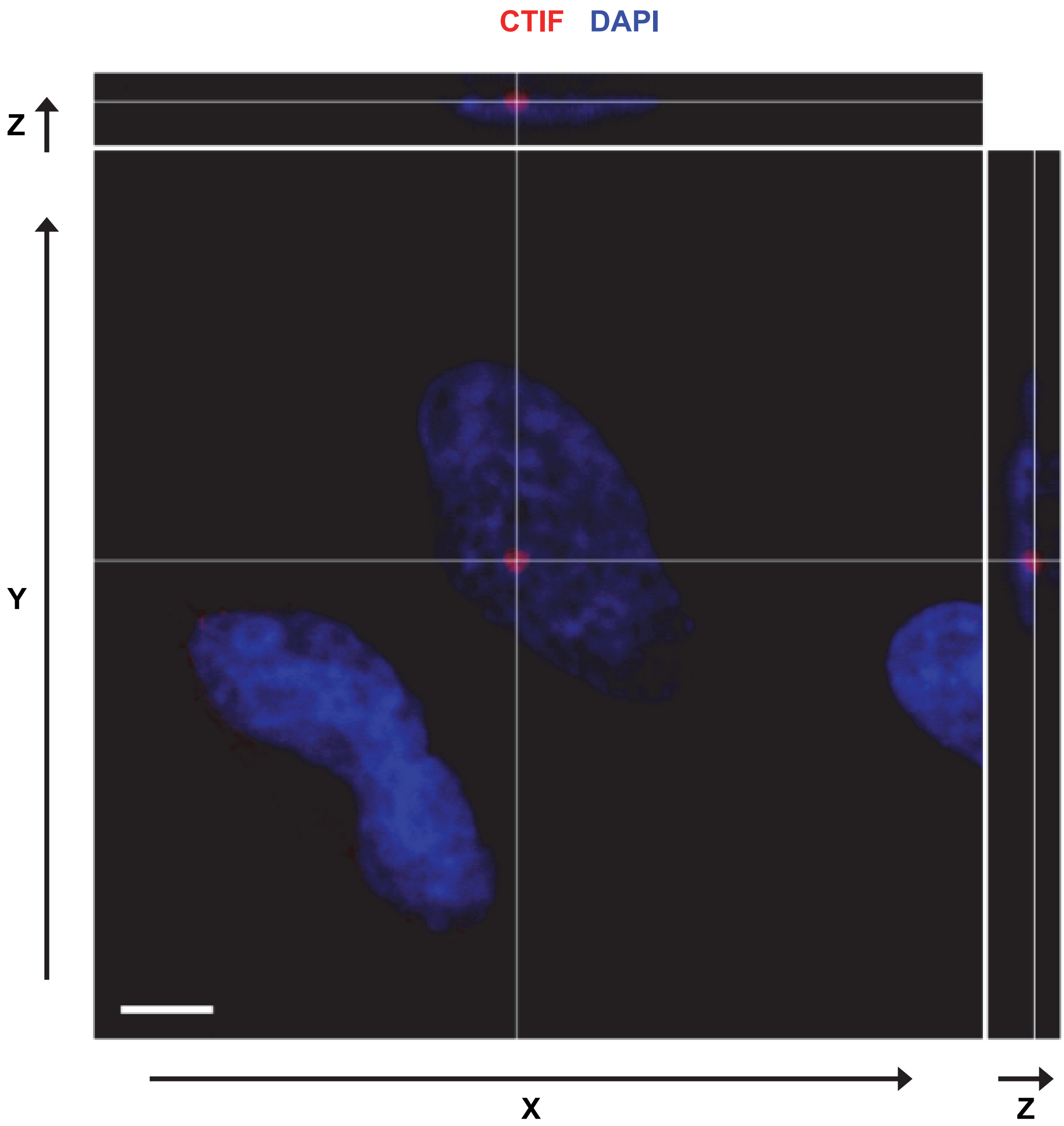
CTIF DAPI
Z
Y
X
Z

Supplementary Figure 1. Three-dimensional (3D) image of a CTIF-enriched cytoplasmic body. A cell exhibiting an aggresome on top of the nucleus was arbitrarily chosen. The 3D image was obtained from a 3D data set collected using a laser scanning confocal microscope. The results are representative of three biological replicates (n = 3). Scale bar, 10 μm.

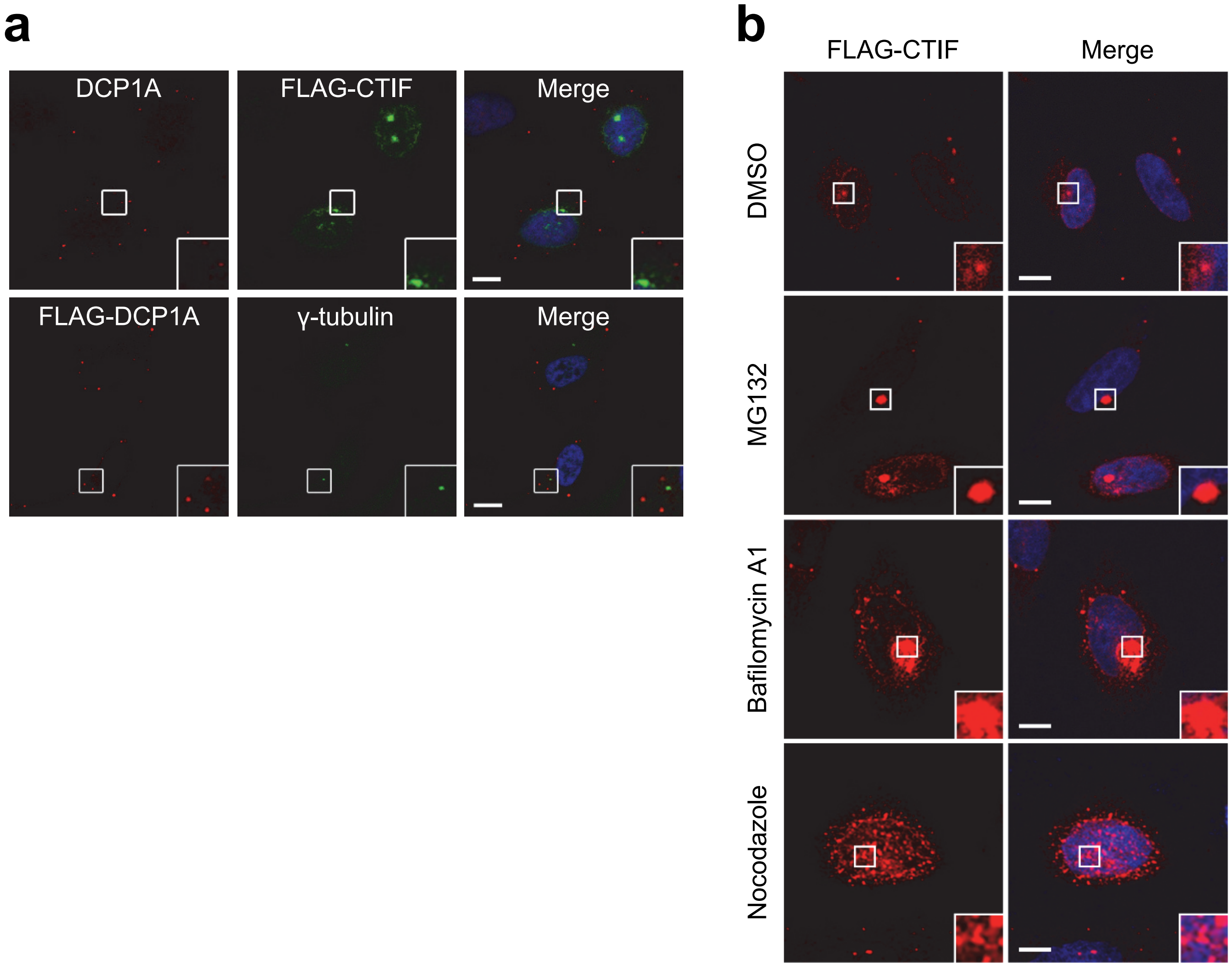
a
DCP1A
FLAG-CTIF
Merge
FLAG-DCP1A
γ-tubulin
Merge
b
FLAG-CTIF
Merge
DMSO
MG132
Bafilomycin A1
Nocodazole

Supplementary Figure 2. FLAG-tagged CTIF is localized to aggresomes. (**a**) Immunostaining of decapping mRNA 1a (DCP1A), FLAG-CTIF, FLAG-DCP1A, and γ-tubulin. (**b**) Immunostaining of FLAG-CTIF (red) in HeLa cells in the presence of the indicated inhibitors. HeLa cells were transiently transfected with a plasmid expressing FLAG-CTIF. One day after transfection, the cells were treated with the indicated inhibitors for 12 h, and then stained using the α-FLAG antibody. n = 3. Scale bar, 10 μm.

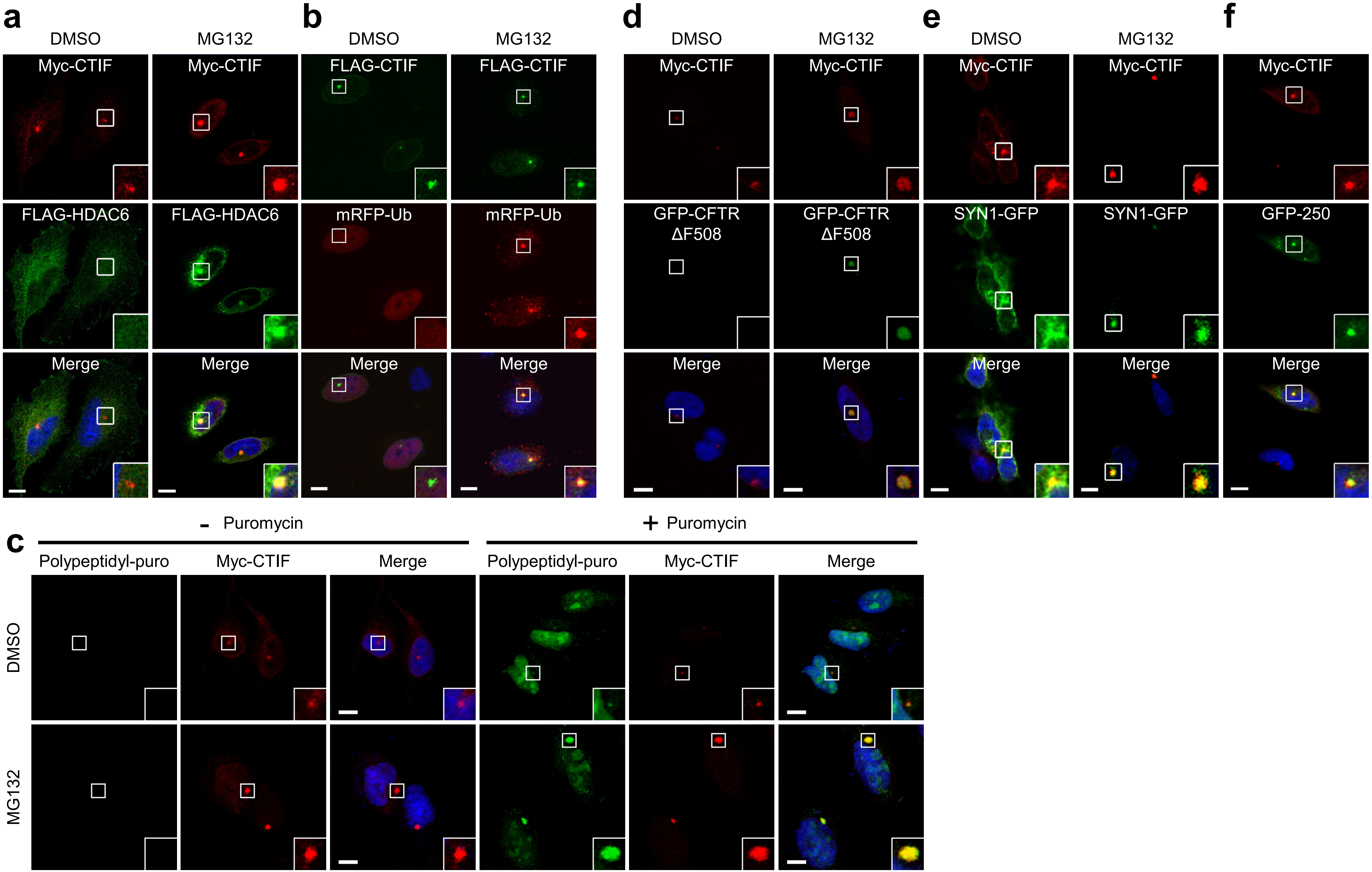
a
DMSO
MG132
Myc-CTIF
FLAG-HDAC6
Merge
b
DMSO
MG132
FLAG-CTIF
mRFP-Ub
Merge
d
DMSO
MG132
Myc-CTIF
GFP-CFTR ΔF508
Merge
e
DMSO
MG132
Myc-CTIF
SYN1-GFP
Merge
f
Myc-CTIF
GFP-250
Merge
c
- Puromycin
+ Puromycin
Polypeptidyl-puro
Myc-CTIF
Merge
DMSO
MG132

Supplementary Figure 3. Immunostaining of CTIF and either previously known aggresomal components or aggresome-targeted misfolded proteins. HeLa cells were transiently transfected with the indicated plasmids. Two days after transfection, the cells were stained using primary antibody against FLAG, Myc, GFP, or puromycin. All results are representative of at least three independent experiments. Where indicated, the cells were treated with DMSO, MG132, or puromycin before cell staining. Scale bar, 10 μm.

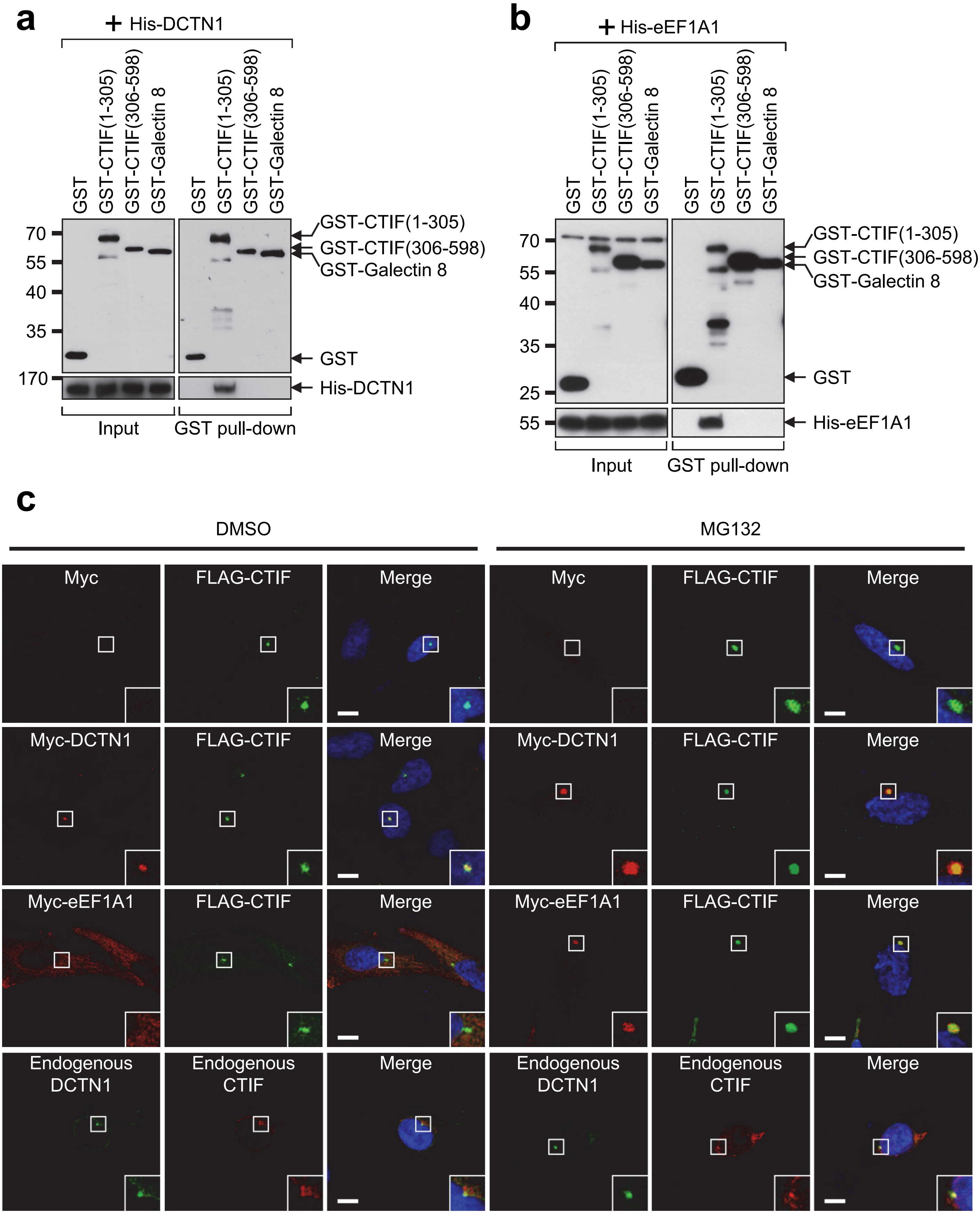
a
+ His-DCTN1
GST
GST-CTIF(1-305)
GST-CTIF(306-598)
GST-Galectin 8
70
55
40
35
170
GST-CTIF(1-305)
GST-CTIF(306-598)
GST-Galectin 8
GST
His-DCTN1
Input
GST pull-down
b
+ His-eEF1A1
70
55
40
35
25
55
His-eEF1A1
c
DMSO
MG132
Myc
FLAG-CTIF
Merge
Myc-DCTN1
Myc-eEF1A1
Endogenous DCTN1
Endogenous CTIF

Supplementary Figure 4. Direct interactions and co-localizations of CTIF with DCTN1 and eEF1A1. (**a**,**b**) GST pull-down assays were performed using recombinant purified GST-fused CTIF variants and either His-DCTN1 (**a**) or His-eEF1A1 (**b**). GST-galectin 8 was used as a negative control. n = 2. (**c**) Immunostaining of CTIF, DCTN1, and eEF1A1. HeLa cells were transiently transfected with the indicated plasmids. One day later, the cells were treated with either DMSO or MG132 for 12 h, and then immunostained using the indicated antibodies. n = 3. Scale bar, 10 μm.

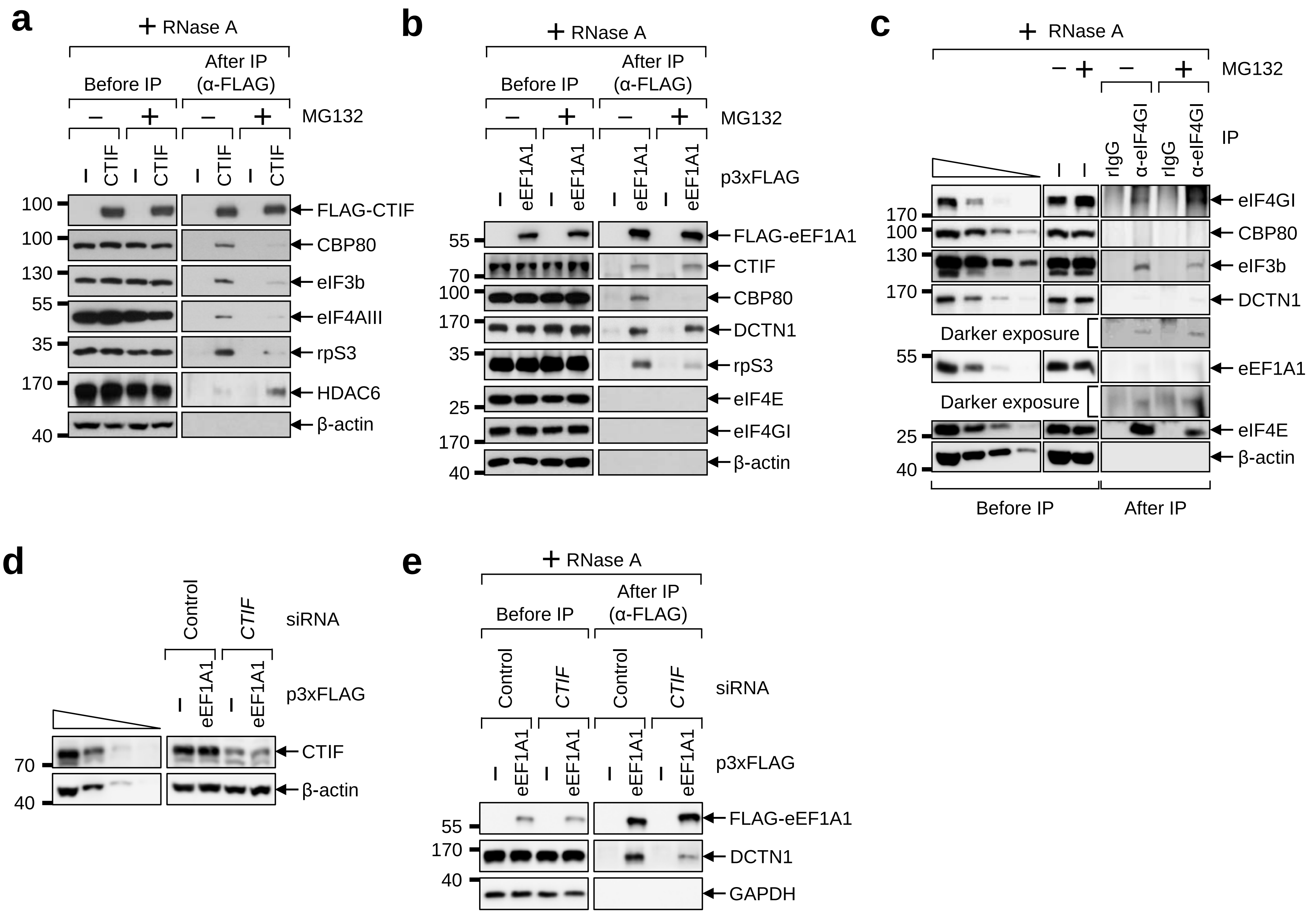

a
+ RNase A
Before IP
After IP (α-FLAG)
− + − +
MG132
CTIF CTIF CTIF CTIF
100
100
130
55
35
170
40
FLAG-CTIF
CBP80
eIF3b
eIF4AIII
rpS3
HDAC6
β-actin
b
+ RNase A
Before IP
After IP (α-FLAG)
− + − +
MG132
p3xFLAG
eEF1A1 eEF1A1 eEF1A1 eEF1A1
55
70
100
170
35
25
170
40
FLAG-eEF1A1
CTIF
CBP80
DCTN1
rpS3
eIF4E
eIF4GI
β-actin
c
+ RNase A
− + − +
MG132
IP
rIgG
α-eIF4GI
rIgG
α-eIF4GI
170
100
130
170
55
25
40
eIF4GI
CBP80
eIF3b
DCTN1
Darker exposure
eEF1A1
Darker exposure
eIF4E
β-actin
Before IP
After IP
d
Control
CTIF
siRNA
eEF1A1
eEF1A1
p3xFLAG
70
40
CTIF
β-actin
e
+ RNase A
Before IP
After IP (α-FLAG)
Control
CTIF
Control
CTIF
siRNA
eEF1A1 eEF1A1 eEF1A1 eEF1A1
p3xFLAG
55
170
40
FLAG-eEF1A1
DCTN1
GAPDH

Supplementary Figure 5. CTIF, eEF1A1, and DCTN1 shift from the CT complex to the aggresomal complex upon MG132 treatment. (**a**) IPs of FLAG-CTIF. As in **Fig. 2b**, except that HEK293T cells were transiently transfected with either pcDNA3-FLAG or pcDNA3-FLAG-CTIF. (**b**) IPs of FLAG-eEF1A1. As in **Fig. 2b**, except that HEK293T cells were transiently transfected with either p3×FLAG or p3×FLAG-eEF1A1. (**c**) IPs of endogenous eIF4GI. As in **Fig. 2b**, except that IPs were performed using either α-eIF4GI antibody or nonspecific rabbit IgG (rIgG). (**d,e**) IPs of FLAG-eEF1A1. As in panel **b**, except that HEK293T cells were either undepleted or depleted of CTIF using siRNA. Specific downregulation was confirmed by Western blotting, as shown in panel **d**. n = 2.

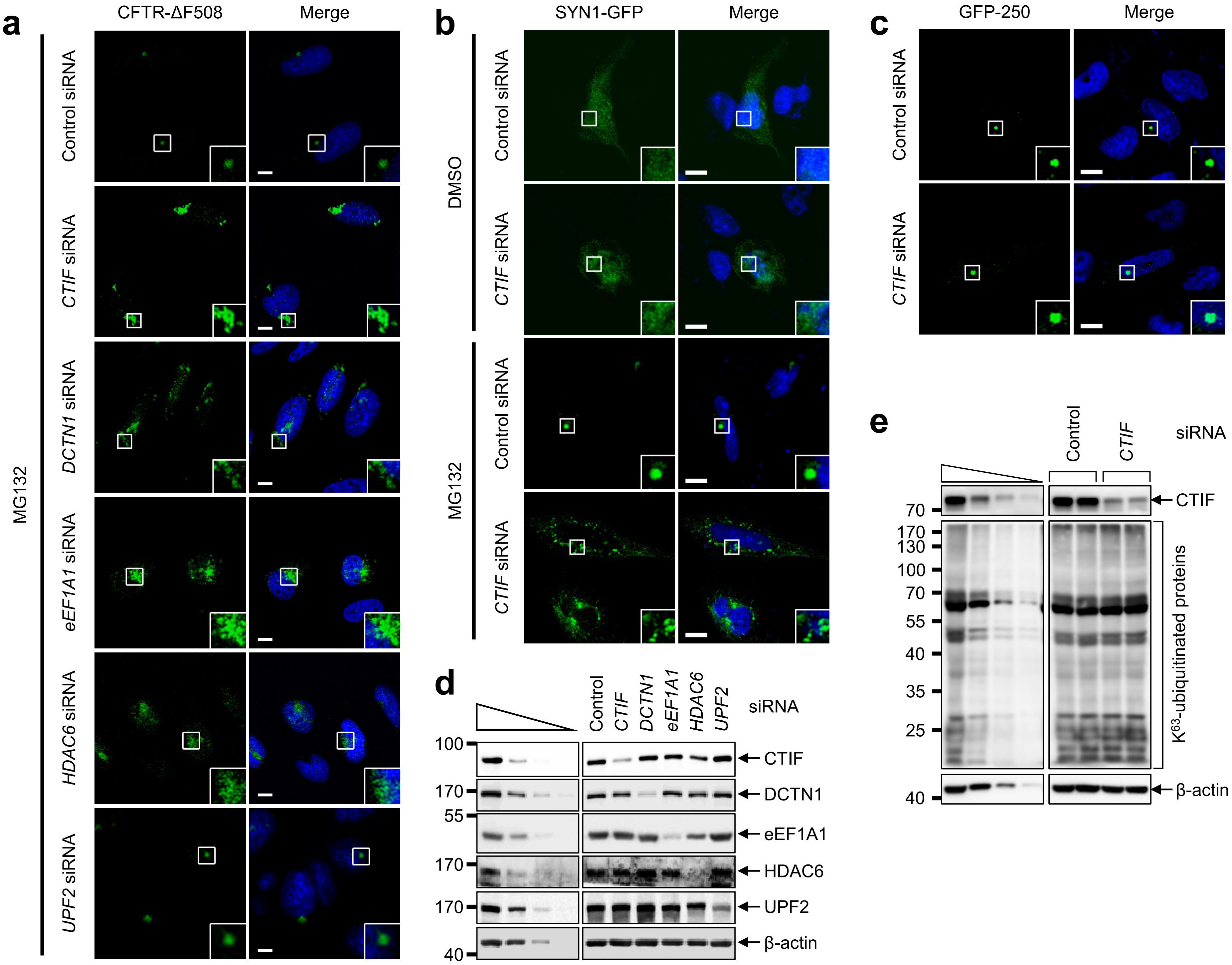

a
CFTR-ΔF508
Merge
MG132
Control siRNA
CTIF siRNA
DCTN1 siRNA
eEF1A1 siRNA
HDAC6 siRNA
UPF2 siRNA
b
SYN1-GFP
Merge
DMSO
MG132
Control siRNA
CTIF siRNA
c
GFP-250
Merge
Control siRNA
CTIF siRNA
d
Control
CTIF
DCTN1
eEF1A1
HDAC6
UPF2
siRNA
100
170
55
170
170
40
CTIF
DCTN1
eEF1A1
HDAC6
UPF2
β-actin
e
Control
CTIF
siRNA
70
170
130
100
70
55
40
35
25
40
CTIF
K63-ubiquitinated proteins
β-actin

Supplementary Figure 6. Aggresomes containing CFTR-ΔF508 and SYN1-GFP, but not GFP-250, are dispersed into small cytoplasmic aggregates by downregulating CED components. (**a**) Immunostaining of CFTR-ΔF508. As in **Fig. 3a**, except that HeLa cells stably expressing CFTR-ΔF508 were immunostained in the presence of MG132. Scale bar, 10 μm. (**b**) Immunostaining of SYN1-GFP. As in **Fig. 3a**, except that transiently expressed SYN1-GFP was immunostained in the absence or presence of MG132. Scale bar, 10 μm. (**c**) Immunostaining of GFP-250. As in **Fig. 3a**, except that transiently expressed GFP-250 was immunostained in the absence of MG132. Scale bar, 10 μm. (**d**) Western blotting demonstrating specific downregulation by siRNA transfection. (**e**) Western blotting showing no significant change in the level of K63-ubiquitinated proteins by CTIF downregulation. All results of immunostainings and IPs are representative of three and two biological replicates, respectively.

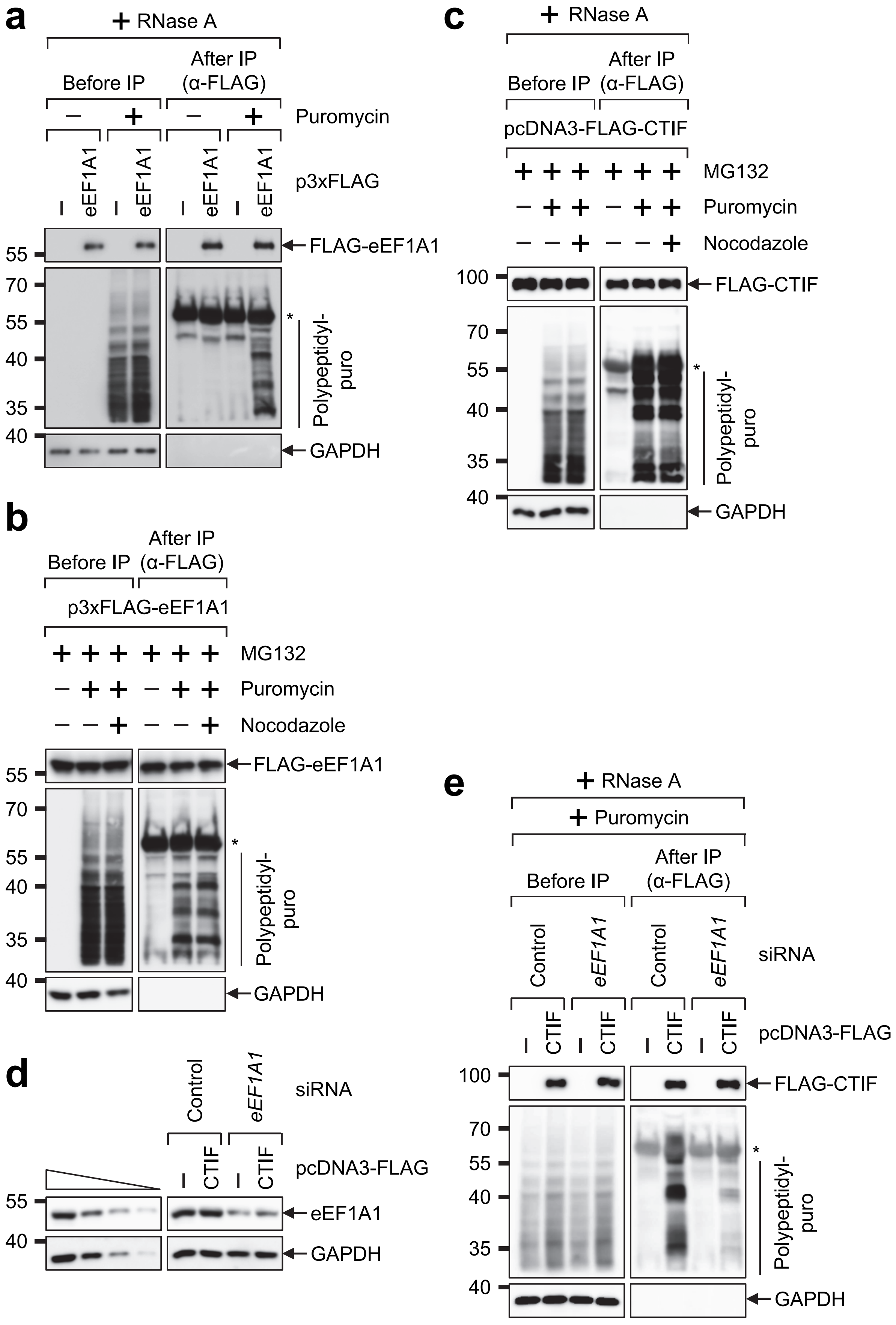
a
+ RNase A
Before IP
After IP (α-FLAG)
Puromycin
p3xFLAG
eEF1A1
FLAG-eEF1A1
GAPDH
Polypeptidyl-puro
55
70
40
35
b
Before IP
After IP (α-FLAG)
p3xFLAG-eEF1A1
MG132
Puromycin
Nocodazole
FLAG-eEF1A1
Polypeptidyl-puro
GAPDH
c
+ RNase A
Before IP
After IP (α-FLAG)
pcDNA3-FLAG-CTIF
MG132
Puromycin
Nocodazole
100
FLAG-CTIF
Polypeptidyl-puro
GAPDH
d
siRNA
Control
eEF1A1
pcDNA3-FLAG
CTIF
eEF1A1
GAPDH
e
+ RNase A
+ Puromycin
Before IP
After IP (α-FLAG)
siRNA
Control
eEF1A1
pcDNA3-FLAG
CTIF
FLAG-CTIF
Polypeptidyl-puro
GAPDH

Supplementary Figure 7. CED complex associates with misfolded polypeptides before aggresome formation. (**a**) IPs of FLAG-eEF1A1. IPs were performed using α-FLAG antibody and extracts of HEK293T cells transiently expressing either FLAG or FLAG-eEF1A1. The cells were either treated or not with puromycin for 1 h before harvesting. Total-cell extracts were treated with RNase A before IP. (**b**) IPs of FLAG-eEF1A1 in the presence of nocodazole. Cells transiently expressing FLAG-eEF1A1 were either treated or not with puromycin for 1 h, MG132 for 12 h, or nocodazole for 12 h before cell harvesting. (**c**) IPs of FLAG-CTIF. As in panel **b**, except that IPs were performed using extracts of HEK293T cells transiently expressing either FLAG or FLAG-CTIF. (**d**,**e**) IPs of FLAG-CTIF using extracts of eEF1A1-depleted HEK293T cells transiently expressing FLAG-CTIF. Immunoglobulin heavy chain is marked with an asterisk (*). Specific downregulation was demonstrated by Western blotting in panel **d**. n = 2.

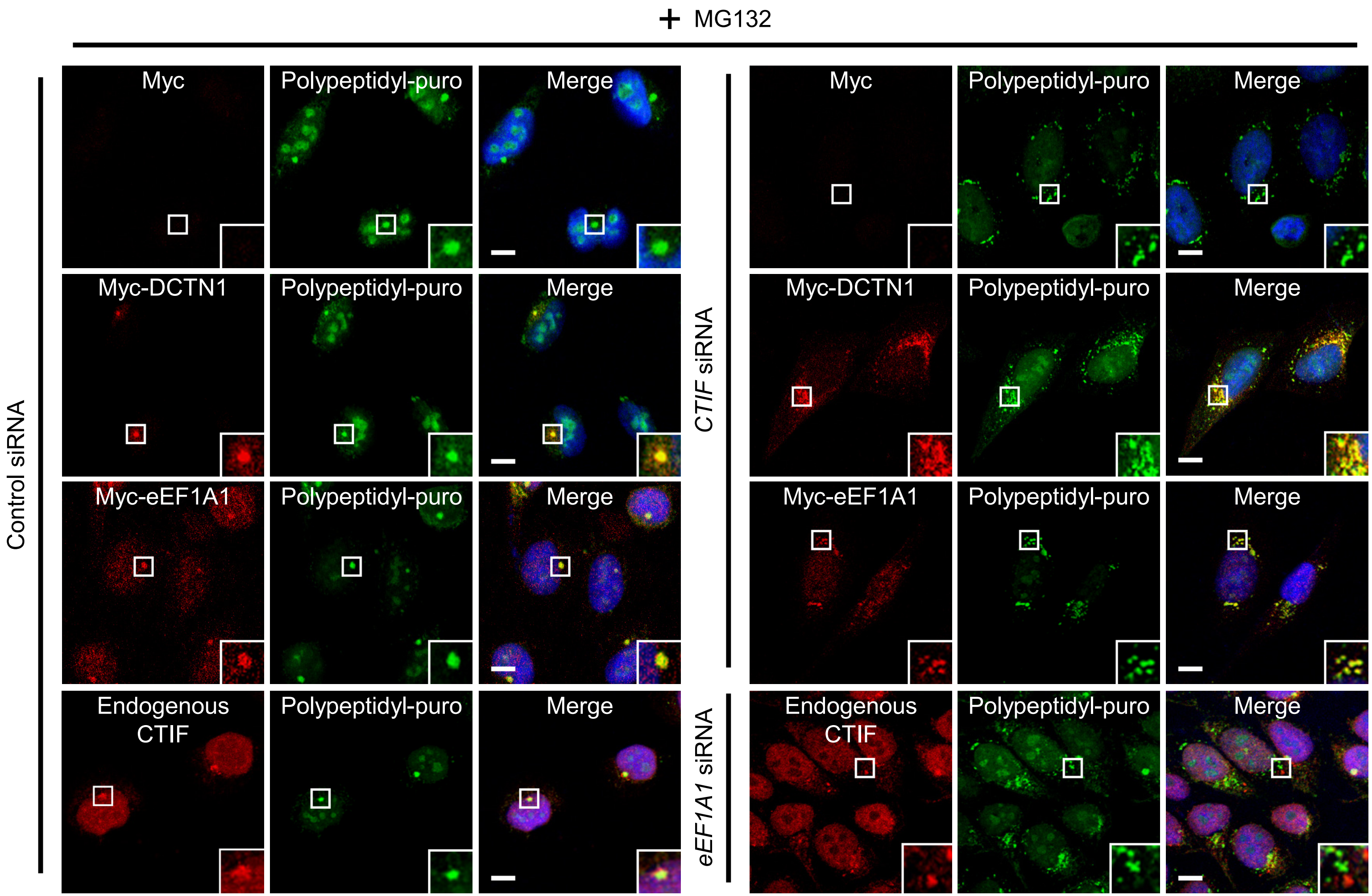

+ MG132
Control siRNA
CTIF siRNA
eEF1A1 siRNA
Myc
Polypeptidyl-puro
Merge
Myc-DCTN1
Myc-eEF1A1
Endogenous CTIF

Supplementary Figure 8. DCTN1 and eEF1A1 are colocalized with small cytoplasmic aggregates containing polypeptidyl-puro. HeLa cells depleted of either CTIF or eEF1A1 were treated with MG132 and puromycin. Transiently expressed Myc-DCTN1 or Myc-eEF1A1 and newly synthesized misfolded polypeptidyl-puro were immunostained using primary antibody against Myc, CTIF, or puromycin. n = 2. Scale bar, 10 μm.

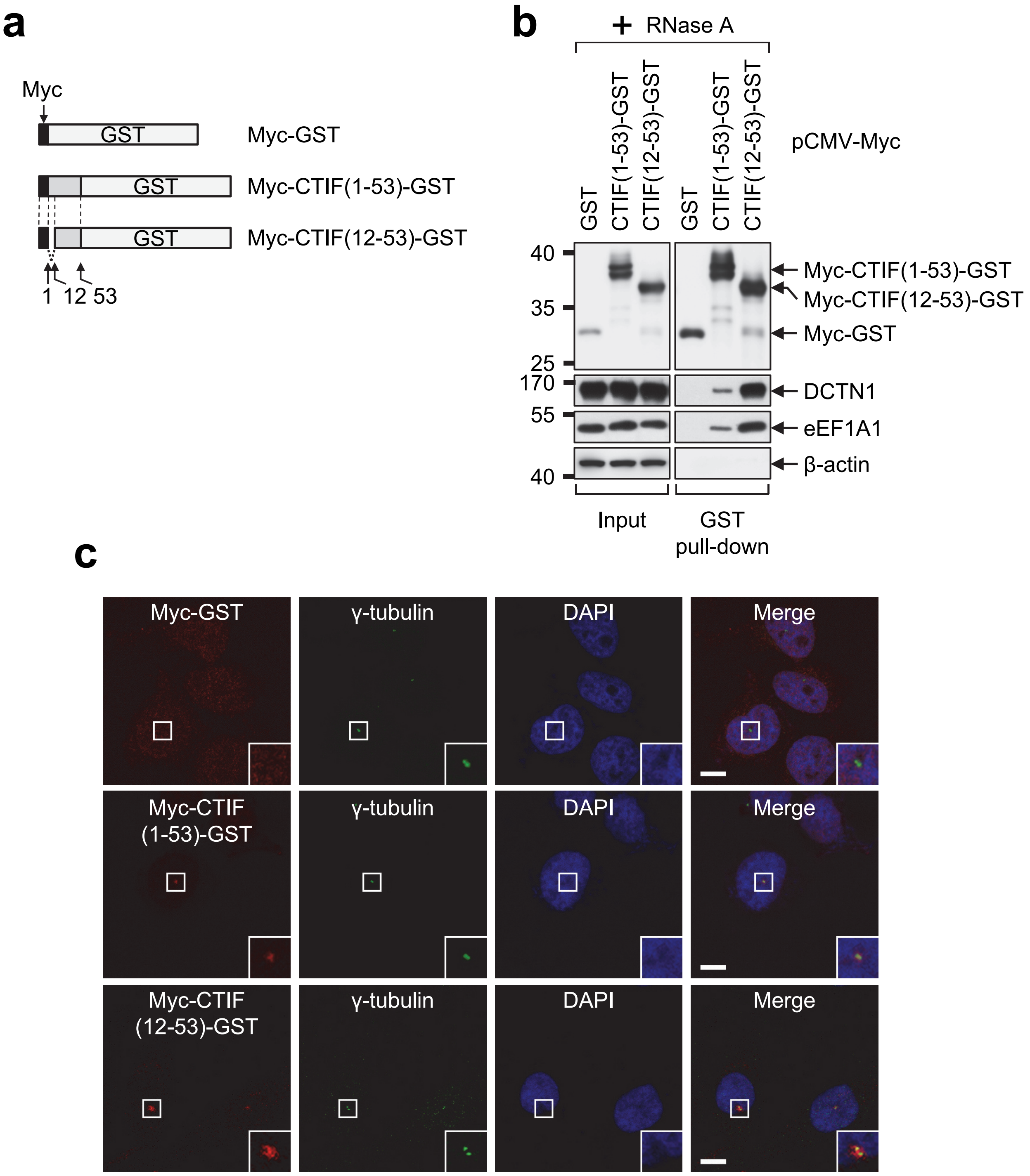
a
Myc
GST
Myc-GST
GST
Myc-CTIF(1-53)-GST
GST
Myc-CTIF(12-53)-GST
1 12 53
b
+ RNase A
GST
CTIF(1-53)-GST
CTIF(12-53)-GST
GST
CTIF(1-53)-GST
CTIF(12-53)-GST
pCMV-Myc
40
35
25
170
55
40
Myc-CTIF(1-53)-GST
Myc-CTIF(12-53)-GST
Myc-GST
DCTN1
eEF1A1
β-actin
Input
GST pull-down
c
Myc-GST
γ-tubulin
DAPI
Merge
Myc-CTIF (1-53)-GST
γ-tubulin
DAPI
Merge
Myc-CTIF (12-53)-GST
γ-tubulin
DAPI
Merge

Supplementary Figure 9. The N-terminal region spanning amino acids 12–53 of CTIF is sufficient for aggresomal targeting. (**a**) Schematic diagram of Myc-GST fusion plasmids in which the cDNA sequences for the N-terminal region spanning amino acids 1–53 or 12–53 of CTIF were inserted between Myc and GST sequences. (**b**) GST pull-down using total-cell extracts of HEK293T transiently expressing Myc-CTIF-GST variants. n = 3. (**c**) Immunostaining for Myc-GST fusion proteins (red) and γ-tubulin (green) in HeLa cells. n = 2. Scale bar, 10 μm.

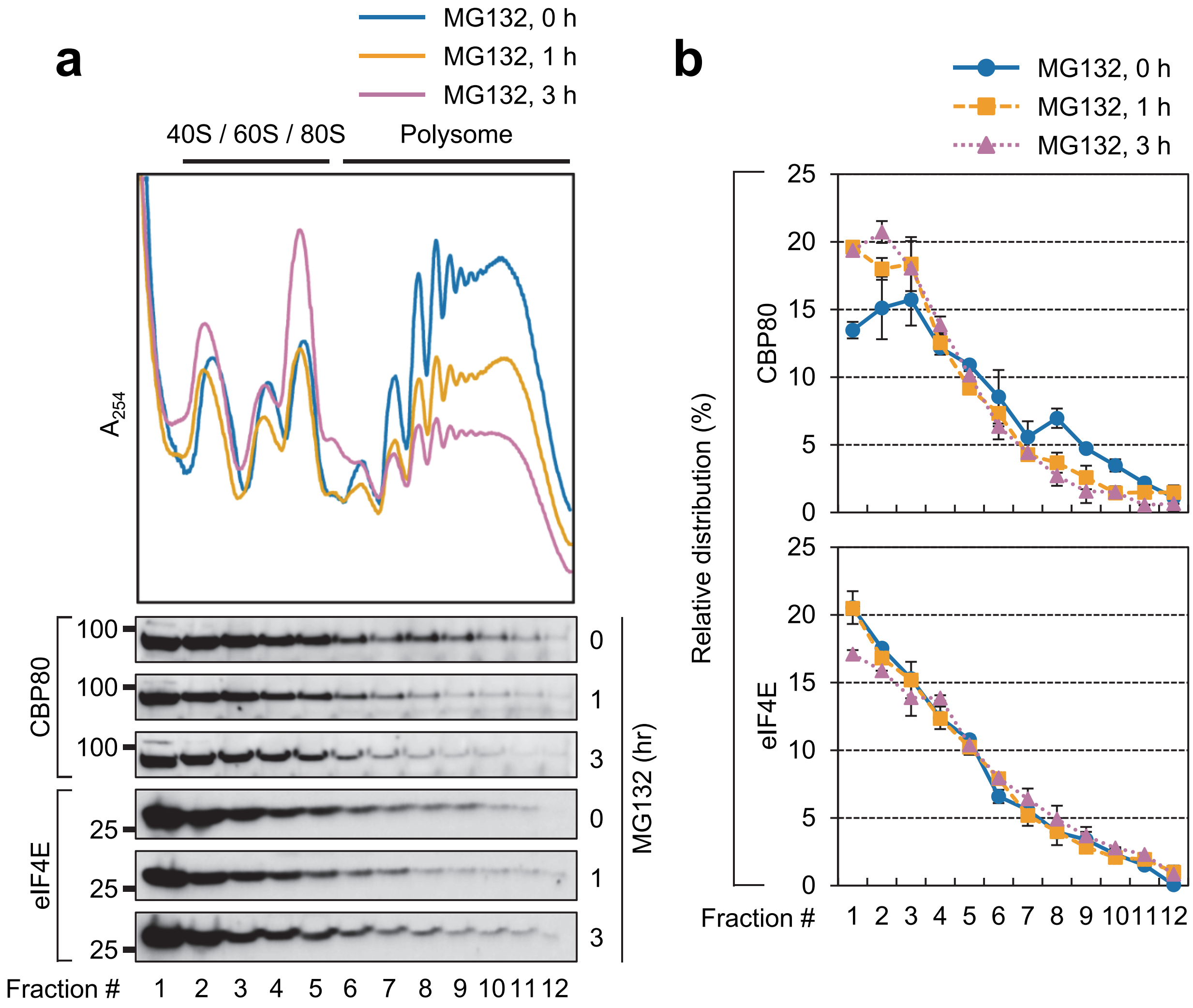
a
MG132, 0 h
MG132, 1 h
MG132, 3 h
40S / 60S / 80S
Polysome
A_{254}
CBP80
eIF4E
100
25
0
1
3
MG132 (hr)
Fraction #
1 2 3 4 5 6 7 8 9 10 11 12
b
MG132, 0 h
MG132, 1 h
MG132, 3 h
Relative distribution (%)
CBP80
eIF4E
0
5
10
15
20
25
Fraction #
1 2 3 4 5 6 7 8 9 10 11 12

Supplementary Figure 10. Treatment of MEF-eIF2α (A/A) cells with MG132 preferentially inhibits CT. (**a**) Polysome fractionation analysis of MEF-eIF2α (A/A) cells. Cells were treated with MG132 for the indicated time. Each fraction was subjected to Western blotting using antibodies against either CBP80 or eIF4E. (**b**) Relative distributions of CBP80 and eIF4E in polysome fractions. The relative levels of CBP80 (upper) and eIF4E (lower) in each fraction were calculated as a percentage of the total. The dots and error bars represent the mean and standard deviation of two biological replicates.

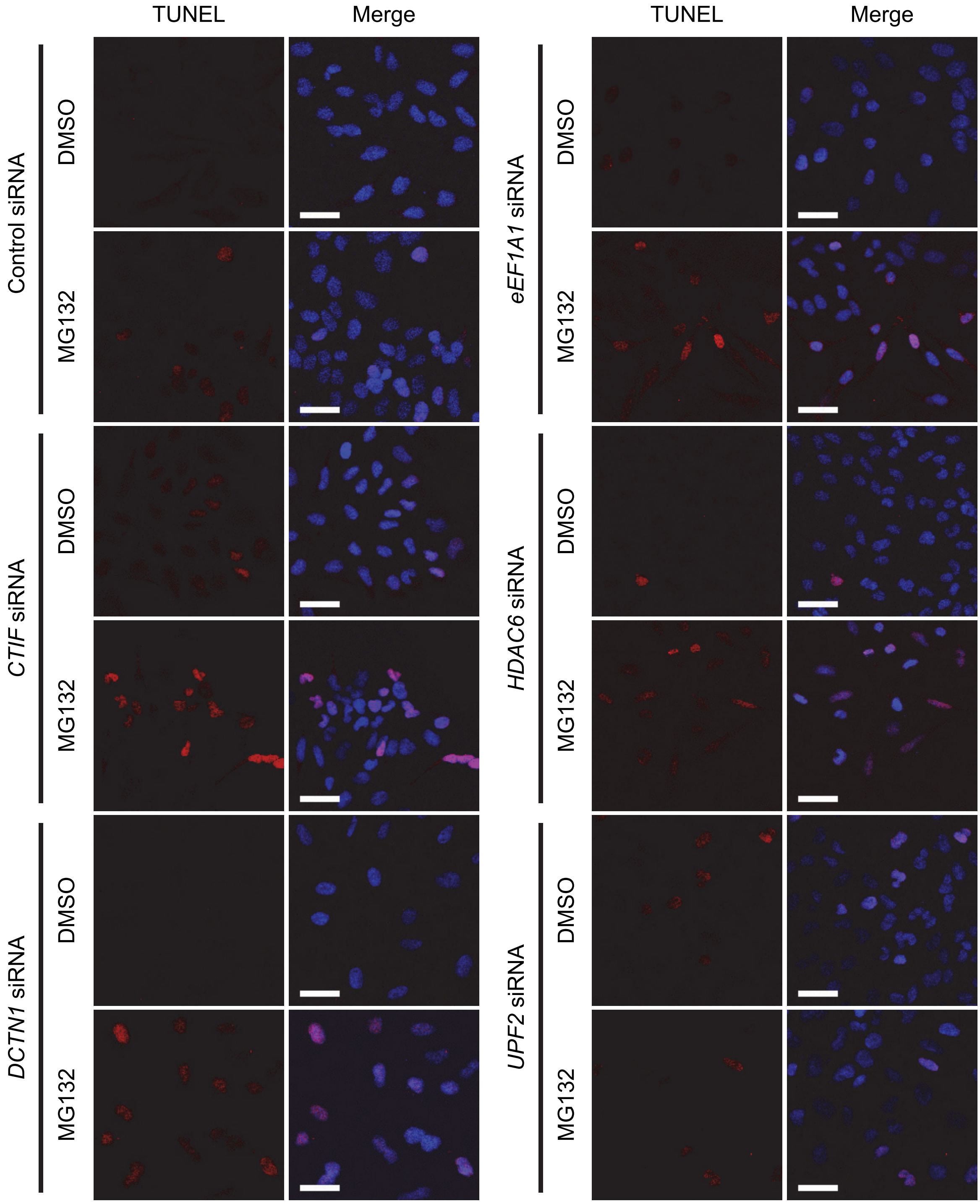
TUNEL
Merge
TUNEL
Merge
Control siRNA
DMSO
MG132
eEF1A1 siRNA
DMSO
MG132
CTIF siRNA
DMSO
MG132
HDAC6 siRNA
DMSO
MG132
DCTN1 siRNA
DMSO
MG132
UPF2 siRNA
DMSO
MG132

Supplementary Figure 11. Downregulation of CED components promotes apoptosis induced by accumulation of misfolded CFTR-ΔF508. TUNEL assay of HeLa cells as described in **Fig. 6a**. n = 3. Scale bar, 50 μm.

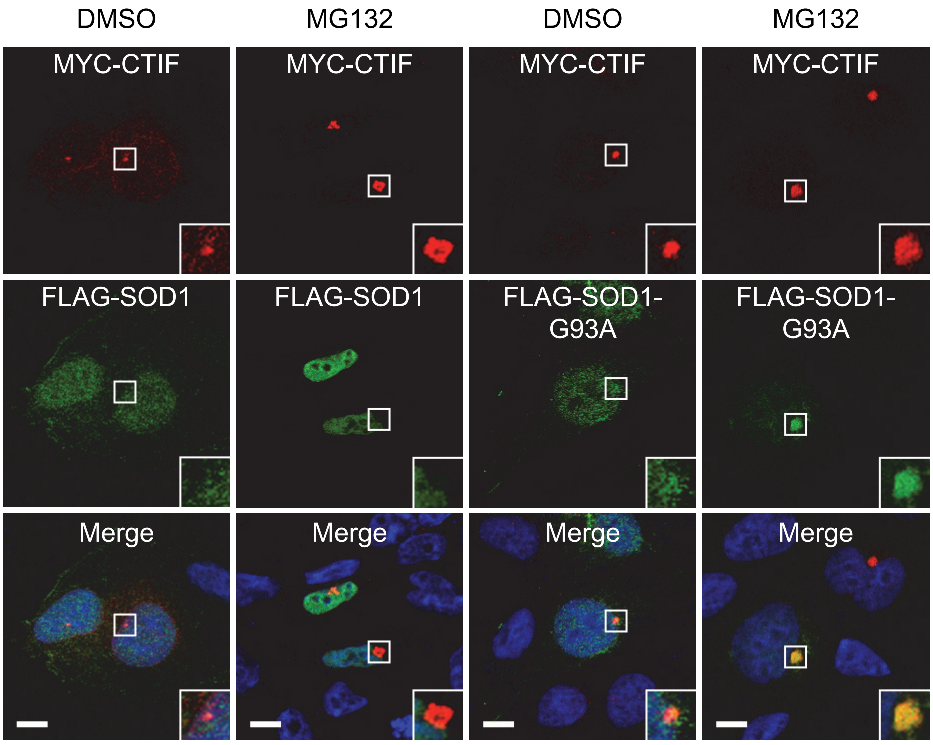
DMSO
MG132
DMSO
MG132
MYC-CTIF
MYC-CTIF
MYC-CTIF
MYC-CTIF
FLAG-SOD1
FLAG-SOD1
FLAG-SOD1-G93A
FLAG-SOD1-G93A
Merge
Merge
Merge
Merge

Supplementary Figure 12. Overlapping of CTIF and SOD1-G93A. Immunostaining of Myc-CTIF and either FLAG-SOD1-WT or -G93A in HeLa cells. n = 2. Scale bar, 10 μm.

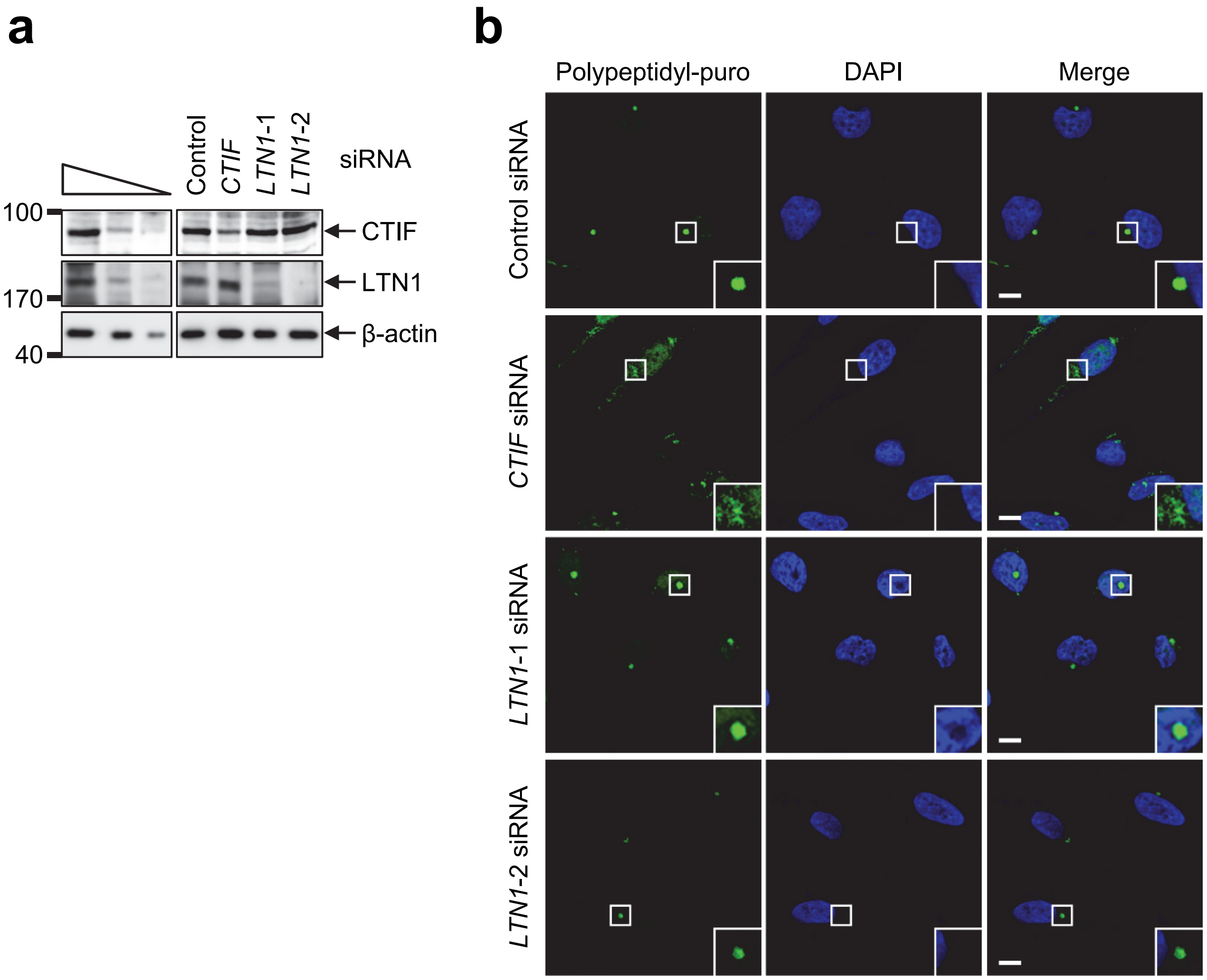

a
b
Control
CTIF
LTN1-1
LTN1-2
siRNA
100
170
40
CTIF
LTN1
β-actin
Polypeptidyl-puro
DAPI
Merge
Control siRNA
CTIF siRNA
LTN1-1 siRNA
LTN1-2 siRNA

Supplementary Figure 13. LTN1 is not involved in CED-mediated aggresome formation. HeLa cells were depleted of either LTN1 or CTIF. The cells were pretreated with MG132 for 12 h and puromycin for 1 h before cell fixing. (**a**) Western blotting demonstrating specific downregulations. (**b**) Immunostaining for polypeptidyl-puro. n = 2. Scale bar, 10 μm.

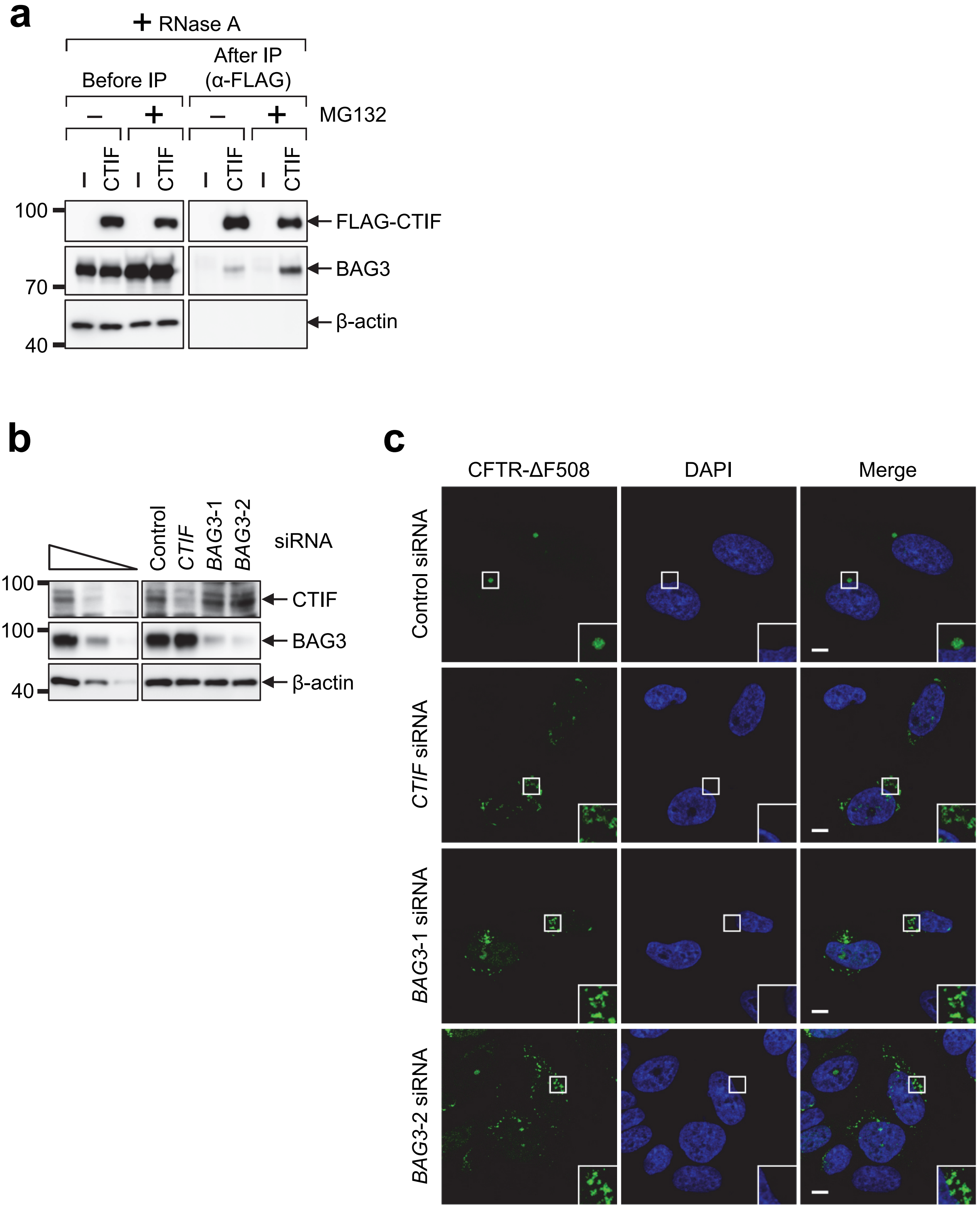
a
+ RNase A
Before IP
After IP (α-FLAG)
MG132
CTIF
100
70
40
FLAG-CTIF
BAG3
β-actin
b
Control
CTIF
BAG3-1
BAG3-2
siRNA
100
100
40
CTIF
BAG3
β-actin
c
CFTR-ΔF508
DAPI
Merge
Control siRNA
CTIF siRNA
BAG3-1 siRNA
BAG3-2 siRNA

Supplementary Figure 14. BAG3 associates with CTIF in a MG132-dependent manner and is involved in the formation of aggresome containing misfolded CFTR-ΔF508. (**a**) IPs of FLAG-CTIF. As in **Fig. 2b**, except that HEK293T cells were transiently transfected with either pcDNA3-FLAG or pcDNA3-FLAG-CTIF. n = 2. (**b,c**) HeLa cells stably expressing CFTR-ΔF508 were depleted of either endogenous BAG3 or CTIF and then immunostained in the presence of MG132. (**b**) Western blotting demonstrating specific downregulations. (**c**) Immunostaining of CFTR-ΔF508. Scale bar, 10 μm. All results of IPs and immunostainings are representative of two and three biological replicates, respectively.

Fig. 2a

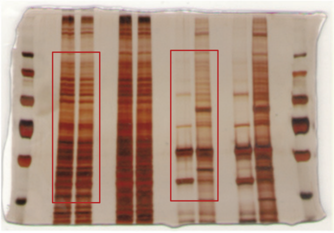

Fig. 2b

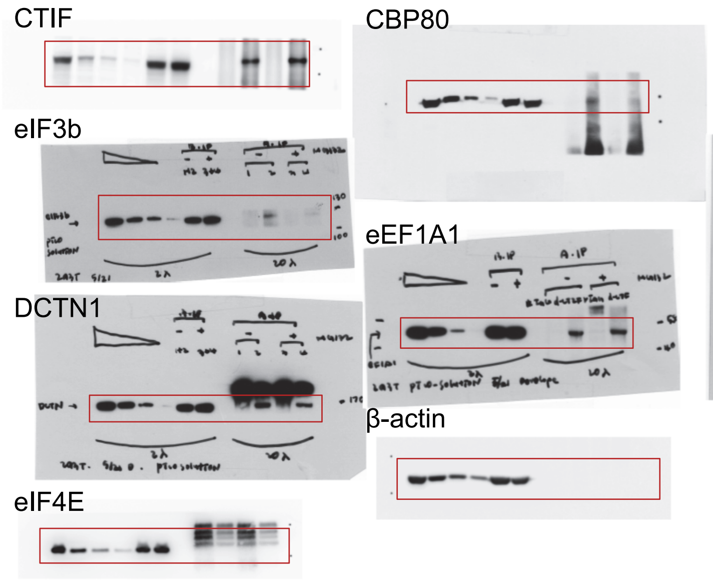

Fig. 2c

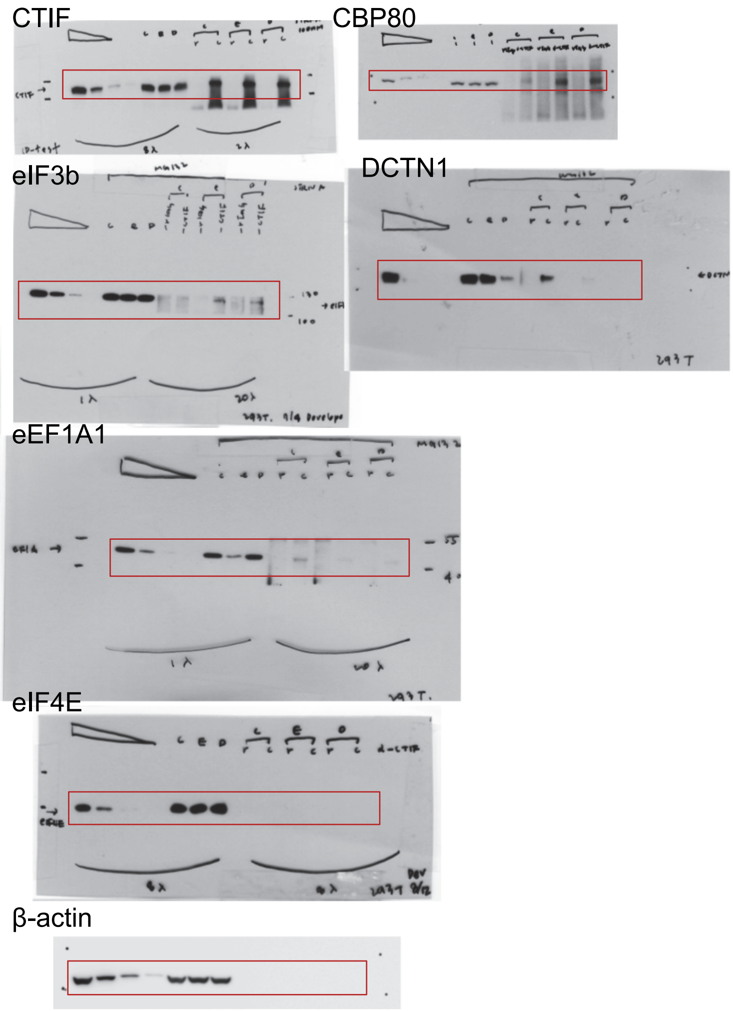

Fig. 2d

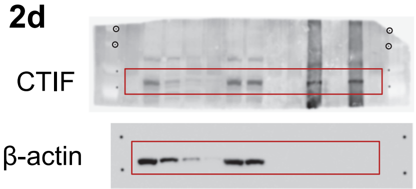

Fig. 4b

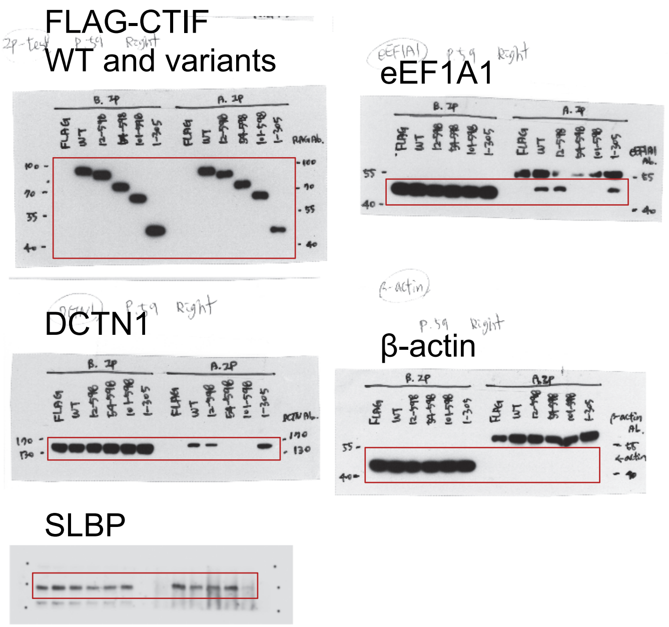

Fig. 5a

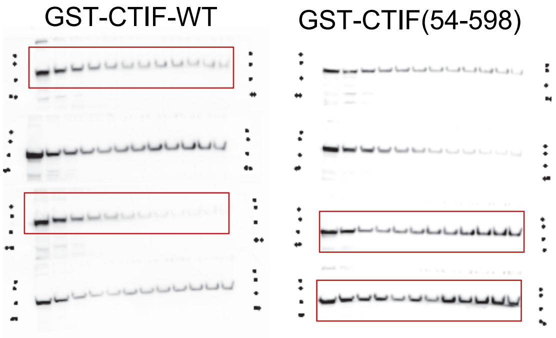

Fig. 6b

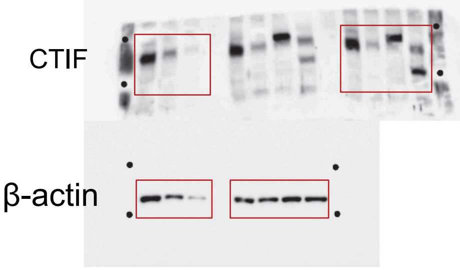

Supplementary Figure 15. Uncropped scans of silver-stained polyacrylamide gels and Western blots.
